# Effects of Angiotensin (1-7) Expressing Lactobacillus and Exercise on Gut-Brain Axis in Aged Rats

**DOI:** 10.1093/geroni/igaa057.3275

**Published:** 2020-12-16

**Authors:** Yi Sun, Youfeng Yang, Anisha Banerjee, Amrisha Verma, Qiuhong Li, Christy Carter, Thomas Buford

**Affiliations:** 1 University of Alabama at Birmingham, Birmingham, Alabama, United States; 2 University of Florida, Gainesville, Florida, United States

## Abstract

Aging is associated with gut dysbiosis – a condition linked with altered central nervous system function (“gut-brain axis”). Age-related health benefits have been ascribed to the renin-angiotensin system, mediated partially via the angiotensin(1-7) axis. Research has shown exercise altering gut microbiota composition and function. This study explored the effects of a genetically modified probiotic expressing angiotensin (1-7) and exercise on the gut-brain axis. Sixty-two male F344/BN rats were randomized at 24-months-old to receive oral gavage of angiotensin (1-7) Lactobacillus paracasei (LP) or LP-A, wide-type LP, or control 3-times/week for 12 weeks; with or without exercise. Rats in exercise groups were walking on a treadmill 10-minutes/day for 5-days/week. Microbiome taxonomic analysis of fecal samples post intervention was performed via 16S-based PCR. A battery of behavior tests were performed before and after the intervention. PCoA revealed that groups differed in the overall fecal microbiota community structure by weighted UniFrac (p=0.034). Indices of alpha-diversity, including Shannon (p=0.02) and Simpson (p=0.019) indexes, displayed LP-A groups with more diverse microbiome than controls. Grip strength (p=0.042) and exercise tolerance (p<0.001) tests showed exercise groups had more muscle strength and aerobic capacity than the sedentary groups. Exercise groups had more lean mass (p=0.019); both LP-A and LP groups had lower fat mass compared to the controls in body composition measurement. LP-A travelled longer distance in the central area than the control in the open field test showing reduced anxiety (p=0.029). Therefore, we conclude LP-A and exercise have distinct and possibly overlapping beneficial effects on the gut-brain axis.

